# Depression and ability to work after vestibular schwannoma surgery: a nationwide registry-based matched cohort study on antidepressants, sedatives, and sick leave

**DOI:** 10.1007/s00701-021-04862-8

**Published:** 2021-05-07

**Authors:** Erik Thurin, Petter Förander, Jiri Bartek, Sasha Gulati, Isabelle Rydén, Anja Smits, Göran Hesselager, Øyvind Salvesen, Asgeir Store Jakola

**Affiliations:** 1 Institute of Neuroscience and Physiology, University of Gothenburg, Sahlgrenska Academy, Box 430, 405 30 Gothenburg, Sweden; 2 Department of Radiology, Sahlgrenska University Hospital, 413 45 Gothenburg, Sweden; 3 Department of Clinical Neuroscience, Karolinska Institute, 17176 Stockholm, Sweden; 4 Department of Neurosurgery, Karolinska University Hospital, 171 76 Stockholm, Sweden; 5 Department of Neurosurgery, Rigshospitalet, Copenhagen, Denmark; 6 Department of Neurosurgery, St., Olavs University Hospital HF, Postboks 3250 Torgarden, 7006 Trondheim, Norway; 7 Department of Neuromedicine and Movement Science, Norwegian University of Science and Technology, Trondheim, Norway; 8 Institute of Neuroscience, Neurology, Uppsala University Hospital, Uppsala, Sweden; 9 Department of Neurosurgery, Uppsala University Hospital, Uppsala, Sweden; 10 Department of Public Health and General Practice, Norwegian University of Science and Technology, Trondheim, Norway; 11 Department of Neurosurgery, Sahlgrenska University Hospital, 413 46, Gothenburg, Sweden

**Keywords:** Vestibular schwannoma, Neurosurgery, Antidepressants, Sick leave

## Abstract

**Background:**

In patients with vestibular schwannomas (VS), tumor control is often achieved, and life expectancy is relatively good. The main risks of surgical treatment are hearing loss and facial nerve function. The occurrence of mood and sleeping disorders in relation to surgery is an important aspect of health that has rarely been studied. Similarly, only limited data exist on the rate of sick leave for patients with VS. In this nationwide registry-based study, we define the use of antidepressants and sedatives and the sick leave pattern before and after VS surgery.

**Methods:**

Adult patients with histopathologically verified VS were identified in the Swedish Brain Tumor Registry (SBTR) and clinical data were linked to relevant national registries after assigning five matched controls to each patient. We studied patterns of dispensed antidepressants and sedative drugs as well as patterns of sick leave compared to respective controls at 2 years before and 2 years following surgery.

**Results:**

We identified 333 patients and 1662 matched controls. The rate of antidepressant use was similar between patients and controls 2 years before surgery (6.0% vs 6.3%) and 2 years after surgery (10.1% vs 7.5%). The rate of sedative use was also similar 2 years before surgery (3.9% vs 4.3%) and 2 years after surgery (4.8% vs 5.3%). The rate of sick leave was similar at baseline between patients and controls, but at 2 years after surgery, 75% of patients vs 88% of controls (*p* < 0.01) had no registered sick leave. Long-term sick leave after surgery was predicted by use of sedatives (OR 0.60, 95% CI 0.38–0.94, *p* = 0.03), more preoperative sick leave (OR 0.91, 95% CI 0.89–0.93, *p* < 0.001), and new-onset neurological deficits after surgery (OR 0.42, 95% CI 0.24–0.76, *p* = 0.004).

**Conclusion:**

This nationwide study shows no significant differences in the use of antidepressants and sedatives between patients and controls, while the rate of postoperative sick leave was higher in patients than in controls after VS surgery. Our findings underpin the importance of avoiding surgical sequelae and facilitating return to normal professional life.

**Supplementary Information:**

The online version contains supplementary material available at 10.1007/s00701-021-04862-8.

## Background

Vestibular schwannomas (VS) are benign slow-growing tumors originating from Schwann cells insulating the eighth cranial nerve [7, 17]. VS causes symptoms like tinnitus, hearing loss, and balance disturbances [21]. However, some VS are found incidentally and never cause symptoms [37]. The main treatments are surgery, stereotactic radiosurgery (SRS), or radiotherapy (RT). Complete surgical tumor removal has a high chance of being curative [35] but may cause hearing loss or facial nerve palsy [3, 34, 36]. SRS also has a high chance of being curative and a smaller risk of facial nerve palsy but is generally recommended for small tumors with a diameter less than 3 cm [19, 22, 29, 45, 48, 49]. Continued observation is recommended in asymptomatic patients without radiological growth [14, 38, 39]. When making treatment decisions for VS patients, it is imperative to have up-to-date knowledge on probable outcomes for the different treatment options. As patients rarely die from a lack of tumor control, mortality and progression-free survival are not sufficiently sensitive to identify the optimal treatment. Therefore, other “softer” outcome measures, such as depression and ability to work, are also of importance. Surveys have been used to study the quality of life after VS surgery, but as these are subjective and associated with many forms of bias, including an often quite problematic response bias [6, 10, 16], it is desirable to complement these with objective methods for measuring patient-centered variables.

Antidepressant use is an objective measure highly correlated to depressive symptoms [9, 13], while the level of postoperative sick leave indicates the extent of disability in surgically treated patients. Only rudimentary data exist on how VS surgery impacts these important aspects. In this study, we describe the use of antidepressants and sedatives, as well as the patterns of sick leave rate, before and after surgical treatment of VS patients compared to a control population.

## Materials and methods

The methods used in this manuscript have been described previously [42]. In short, we have combined data for each patient from several nationwide Swedish registries, linked through the unique personal identification numbers given to all citizens. We have used this approach to compare the rate of antidepressant use, sedative use, and sick leave between patients with VS and matched controls. Details regarding patient selection, the included registries, and the statistical analyses are presented below. Definitions of variables are available in Table 1.

**Table 1:** Definition of variables

Variable	Definition	Source of information
Index date	Date of surgery for patients. Controls received the same index date as their respective cases	SBTR
Educational level	Higher education was defined as having any registered completed secondary or tertiary education (college or university)	Statistics Sweden
Disposable income	The sum of total personal yearly income (including salary, child-, and housing allowance) minus tax, in Swedish Crowns (SEK)	Statistics Sweden
Return to work	Assumed to have occurred if no longer receiving compensation. Return could be partial (25, 50, or 75%) or complete (100%)	Swedish Social Insurance Agency
Net days absent	A construct of days and grade of compensation where days multiplied with a degree of compensation created a value between 0 and 365 in a year	Swedish Social Insurance Agency
History of depression	ATC: N06A and/or “depression” according to Elixhauser comorbidity index (F20.4, F31.3-F31.5, F32, F33, F34.1, F41.2, F43.2)	NPR and NPrR
History of seizure	Any previous prescription of ATC: N03A and/or ICD-10: G40. Also, if registered “seizure” as a symptom in SBTR	NPR, NPrR, and SBTR
Elixhauser comorbidity index	According to the index, depression was removed as it is reported separately. The following conditions were removed from the index due to possible association with diagnosis of VS: *C70-72: Malignant tumor in the central nervous system* Using this index, both cases and controls received a score of comorbid categories. We report as 0, 1, 2, or ≥ 3 categories present	NPR
Prescription group	All drugs with a common ATC code. Groups were defined as follows: Antidepressants: ATC class N06A (antidepressants) Sedatives: ATC class N05B (anxiolytics, including benzodiazepines) and N05C (hypnotics and sedatives)	NPrR
Active use	Active use of a prescription group was defined as having received any prescription of a drug of this prescription group in the prior 90 days For the prescription group “sedatives,” the patient was considered an active user only for 30 days after a drug prescription When calculating the percentage of the population that is active users, only alive individuals were considered	NPrR

*ATC*, anatomical therapeutic chemical; *SBTR*, Swedish brain tumor registry; *NPR*, national patient registry; *NPrR*, national prescription registry

### Patient selection

We identified 346 patients in the Swedish Brain Tumor Registry (SBTR) surgically treated for a histopathological verified (according to the 2007 WHO classification) VS between April 1, 2009, and December 31, 2015. No duplicate cases were found, and all patients were ≥ 18 years old. One patient who did not have a registered age was excluded. For each of the six regions providing information to the registry, we required an annual registration rate of ≥ 80% compared to the compulsory national cancer registry for the data to be included, resulting in the exclusion of 12 patients. The total cohort consisted of 333 patients. To study the impact of VS surgery on sick leave patterns, a subpopulation of patients within working age was defined. For this purpose, 90 patients with age ≥ 61 years (of the main cohort with 333 patients) were excluded from the subpopulation since age > 60 was considered to interfere with the decision to retire [42]. Of the remaining 242 patients, we excluded another 36 patients from the subpopulation that were not on registered sick leave on the day of surgery. As described previously [32, 42], this indicated that those patients were detached from the Swedish sick leave system and did not consistently receive compensation for sick leave. The remaining 206 patients constituted the return to work cohort (RTW cohort). See Table 1 for definitions. Every patient was matched to five unique controls, using matching criteria described below, yielding a control population of 1662 controls, of which 1025 were controls for the RTW cohort. One control registered as deceased, but without a registered date of death, it was excluded. For five patients, an incorrect number of controls was identified. See Supplementary Fig. 1 for a flowchart of patient selection.

**Fig. 1:**
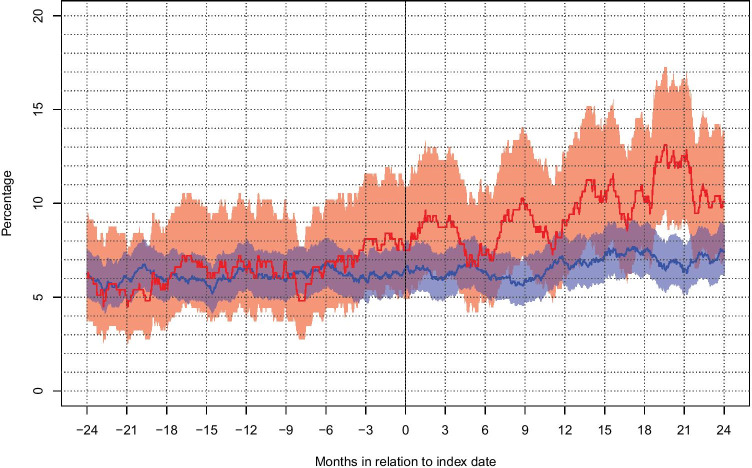
Graph representing the proportions (95% CI) of patients (red) and controls (blue) with active use of antidepressants 2 years prior to the index date through 2 years following the index date

### Linking of registries

The linkage of SBTR to other national registries has been described in our previous studies [32, 42]. In short, SBTR is a nationwide, but regionally based, registry of adult (≥ 18 years) patients with primary brain tumors, with a surgically acquired histopathological diagnosis. SBTR data was linked to several national registries: the patient registry and the prescription registry (part of National Board of Health and Welfare), the Register of the Total Population (part of Statistics Sweden), and the Swedish Social Insurance Agendy: MiDAS database. Statistics Sweden assigned the matched control population, where year of birth, sex, municipality of residence, and educational level were accounted for. All controls were unique. ICD-10 codes from the patient registry were used to calculate comorbidity according to the Elixhauser comorbidity index [8, 26]. From the prescription registry, we received information concerning the type of drug according to the Anatomical Therapeutic Chemical (ATC) classification system and date of dispensing, for the period 2007–2017. Drug groups were defined using the ATC system. The group of antidepressants consisted of all drugs of the N06A (antidepressant) class. The sedative group consisted of all drugs of the N05B (anxiolytics) and N05C (hypnotics and sedatives) classes.

### Statistics

In short, data from the registries were imported into corresponding tables in a mySQL database. Data on drug prescriptions (date and ATC code) and sick leave (dates and degree) were individually analyzed for each patient and combined with clinical data using Python. Definitions regarding the prescription groups and regarding active use are provided in Table 1. Other data derivations were done using mySQL. R was used for statistical analyses. The index date and date of diagnosis were defined as the date of surgery. For each day from 2 years (730 days) before until 2 years after the index date, we calculated the proportion of patients and controls that were active users of the different prescription groups and the proportion of patients and controls that were on full sick leave, partial sick leave, or without any sick leave. The proportions were displayed as graphs. Analyzing Elixhauser comorbidity, we excluded conditions associated with VS, as defined in Table 1 [8, 26].

Other data derivations were done using mySQL. R version 2.13.1 was used for statistical analyses. Continuous variables were summarized using the median, first, and third quartiles, and compared between cases and controls using the Mann–Whitney *U* test. Categorical variables were summarized using counts and proportions and compared between cases and controls using Fisher’s exact test.

Univariable and multivariable logistic regressions were used to examine predictors for RTW (partial or complete) at the end of the follow-up at 2 years postoperatively. Variables were selected based on presumed relevance.

## Ethics statement

This study was approved by the regional ethical committee in the Västra Götaland region (Dnr: 363–17).

## Results

### Demographic data

Baseline characteristics of the 333 included patients are presented in Table 2. The median age was 52 years and 48% of the population was females. The majority of patients (85%) could perform at least light work (i.e., WHO functional status 0–1) at the time of surgery. The preoperative neurologic deficit, including auditory symptoms, was present in 87% of patients, while 7% were asymptomatic preoperatively. Most patients (86%) had tumors measuring < 4 cm, only four patients (2%) had tumors measuring > 6 cm. Gross total resection was achieved in 68%, near-total resection in 7%, and partial resection in 26% of patients.

**Table 2:** Baseline and treatment characteristics for patients with vestibular schwannoma

Variable	RTW cohort (*n* = 206)	All patients (*n* = 333)
Age, median (Q1, Q3)	46 (36, 53)	52 (40, 61)
Female, *n* (%)	91 (44.2)	161 (48.3)
WHO functional status, *n* (%)		
0, fully active	144 (71.0)	210 (64.4)
1, light work possible	39 (19.2)	68 (20.9)
2, cares for self	18 (8.9)	41 (12.6)
3, limited self-care	1 (0.5)	6 (1.8)
4, disabled, confined to bed	1 (0.5)	1 (0.3)
Missing	3	7
Asymptomatic preoperatively, *n* (%)	12 (5.8)	23 (6.9)
Neurologic deficit preoperatively, *n* (%)	178 (86.4)	288 (86.5)
Tumor laterality, *n* (%)		
Left	100 (51.3)	162 (47.9)
Right	94 (48.2)	151 (51.4)
Bilateral	1 (0.5)	2 (0.6)
Missing	11	18
Tumor size, *n* (%)		
< 4 cm	149 (85.6)	244 (85.9)
4–6 cm	21 (12.1)	36 (12.7)
> 6 cm	4 (2.3)	4 (1.4)
Missing	32	49
Extent of resection, *n* (%)		
Partial resection	53 (25.7)	93 (27.9)
Near total resection	14 (6.8)	18 (5.4)
Total resection	139 (67.5)	222 (66.7)
New deficit after surgery, *n* (%)	58 (28.2)	93 (27.9)
Postoperative complications		
Infection, *n* (%)	25 (12.1)	34 (10.2)
Hemorrhage, *n* (%)	7 (3.4)	10 (3.0)
Venous thromboembolism, *n* (%)	1 (3.9)	10 (3.0)
Seizure, *n* (%)	0	0
Reoperation due to complication, *n* (%)	15 (7.3)	21 (6.3)
Oncological treatment planned, *n* (%)	8 (3.9)	9 (2.7)
Missing	1	1

### Patterns of antidepressant and sedative use

**Fig. 2:**
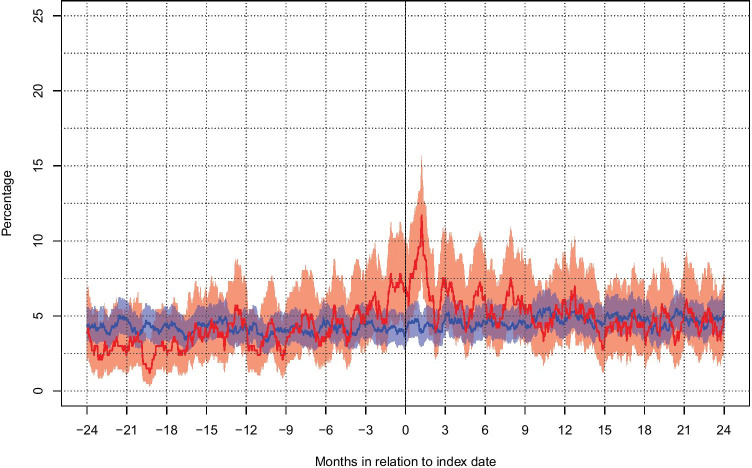
Graph representing the proportions (95% CI) of patients (red) and controls (blue) with active use of sedatives 2 years prior to the index date through 2 years following the index date

A comparison between all VS patients (*n* = 333) and all controls (*n* = 1662) regarding socioeconomic variables, comorbidity, and patterns of antidepressant and sedative use is summarized in Table 3. The percentage of patients and controls with active use of each of the defined drug groups is presented at 2 years before until 2 years following index date in Figs. 1 and 2. There were no significant differences between patients and controls regarding the use of antidepressants and sedatives at the index date, at 2 years prior to surgery, or at 2 years following surgery. As seen in Fig. 2, the use of sedatives among patients was significantly higher than for controls around 1–2 months after surgery but then dropped to the level of the controls.

**Table 3:** Characteristics of patients and controls concerning socioeconomic factors and use of mood-related medications

	All patients (*n* = 333)	All controls (*n* = 1662)	*p*-value
Educational level, at the index year, *n* (%)			
Basic to high school	208 (63.4)	1030 (62.9)	0.86
Higher education	120 (36.6)	607 (37.1)	
Missing	5	253	
Disposable income, 1000 SEK			
Median (Q1, Q3)	218 (158, 286)	231 (154, 310)	0.19
Elixhauser comorbidities, at the index date, *n* (%)			
0	276 (82.9)	1446 (87.0)	
1	39 (11.7)	144 (8.7)	0.23
2	12 (3.6)	44 (2.6)	
3 or more	6 (1.8)	28 (1.7)	
Use of antidepressants at 2 years before the index date, *n* (%)	20 (6.0)	104 (6.3)	1.0
Use of sedatives at 2 years before the index date, *n* (%)	13 (3.9)	72 (4.3)	0.88
Use of antidepressants at the index date, *n* (%)	25 (7.5)	107 (6.4)	0.64
Use of sedatives at the index date, *n* (%)	23 (6.9)	64 (3.8)	0.08
Use of antidepressants at 2 years after the index date, *n*/*n* alive (% of alive)	33/327 (10.1)	122/1637 (7.5)	0.15
Use of sedatives at 2 years after the index date, *n*/*n* alive (% of alive)	16/327 (4.8)	87/1637 (5.3)	0.85

### Return to work

**Fig. 3:**
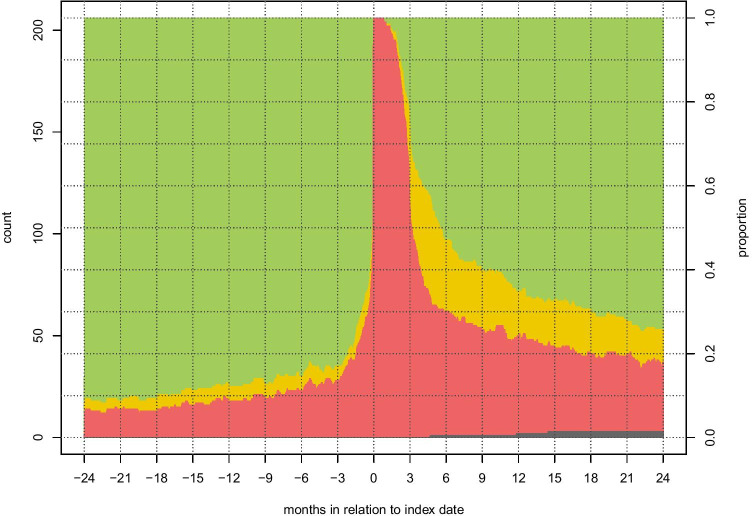
Stacked graph demonstrating, in patients with vestibular schwannoma (*n* = 206), the rates without sick leave compensation (green), with partial compensation (yellow), and with full compensation (red) from 365 days prior to the index date to 730 days after the index date. The dark gray stack at the bottom represents deceased patients

**Fig. 4:**
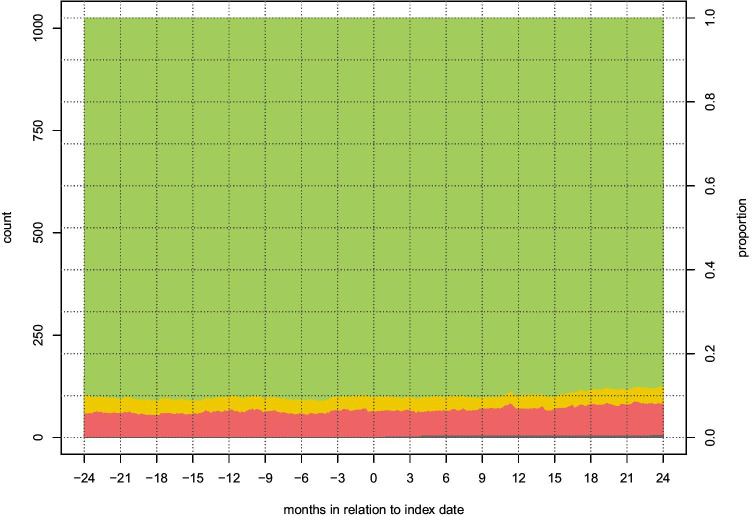
Stacked graph demonstrating, in matched controls (*n* = 1025), the rates without sick leave compensation (green), with partial compensation (yellow), and with full compensation (red) from 365 days prior to the index date to 730 days after the index date. The dark gray stack at the bottom represents deceased patients

The RTW cohort (*n* = 206) and respective controls (*n* = 1025) are summarized regarding patterns of sick leave in Table 4. Absence from work in the 2 years preceding surgery up to 2 years following surgery for the RTW cohort is presented in separate graphs for patients (Fig. 3) and controls (Fig. 4).

**Table 4:** Subgroup analysis: quantification of postoperative disability among vestibular schwannoma patients in the workforce, compared to their controls

	RTW cohort (*n* = 206)	Controls (*n* = 1025)	*p*-value
Net days absent 365 days prior to the index date, median (Q1, Q3)	5 (1, 53)	0 (0, 0)	< 0.01
% on *permanent* sick leave at the index date	13 (6.3)	42 (4.1)	0.64
Net days absent 1 year after the index date, median (Q1, Q3)	136 (89, 329)	0 (0, 0)	< 0.01
Net days absent between 1 and 2 years after the index date, median (Q1, Q3)	0 (0, 140)	0 (0, 0)	< 0.01
Without any sick leave compensation at 2 years before the index date, *n* (%)	188 (91.2)	924 (90.1)	0.62
Without any sick leave compensation at 1 year before the index date, *n* (%)	181 (87.9)	926 (90.3)	0.46
Without any sick leave compensation at 1 year after the index date, *n* (%)	137 (66.5)	927 (90.4)	< 0.01
Without any sick leave compensation 2 years after the index date, *n* (%)	155 (75.2)	907 (88.5)	< 0.01
On *permanent* sick leave 365 days after the index date, *n* (%)	13 (6.3)	40 (3.9)	0.35

At 1 year before surgery, 88% of patients did not have any registered sick leave. The rate of complete RTW (no sick leave) among patients was 25% at 3 months, 53% at 6 months, 67% at 1 year, and 75% at 2 years after surgery. The rate of controls without any registered sick leave was stable at 88–92% during the studied time.

### Predictors for returning to work

To establish predictors for RTW at 2 years after VS surgery, a logistic regression model was created (Supplementary Table 1). We found that net days absent in the year preceding surgery (OR 0.91, 95% CI 0.89–0.93, *p* < 0.001), use of sedatives at the index date (OR 0.60, 95% CI 0.38–0.94, *p* = 0.03), and surgically acquired neurological deficits (OR 0.42, 95% CI 0.24–0.76, *p* = 0.004) were all negatively associated with RTW at 2 years after surgery.

## Discussion

In this nationwide register-based study, we demonstrated that the rates of antidepressant and sedative use were not significantly different for patients 2 years after VS surgery compared to matched controls, but only 75% of patients were in full-time employment, compared to 88% of matched controls 2 years after surgery. Predictors for being on sick leave 2 years after surgery were sedative use at the index date, more preoperative sick leave, and new-onset neurological deficits after surgery.

### Drug use

The main indication for antidepressants is major depressive disorder (MDD). Antidepressants are also prescribed for other conditions with partially overlapping diagnostic criteria, such as anxiety disorders and sleep disorders, and use is increasing [24]. The rate of antidepressant use is tightly correlated to the severity of depressive symptoms [9] and has been utilized to indicate the prevalence of depression [13].

In the present study, the use of antidepressants was similar between cases and controls at 2 years before surgery, at the index date, and at 2 years after surgery. This is in line with a previous cross-sectional study of 205 VS patients using validated questionnaires to measure symptoms of depression and anxiety at different time points after diagnosis or treatment for VS, concluding that anxiety and depression scores did not differ from the general population [4]. The same study also reported that surgical management, radiation, and observational management were associated with anxiety and depression scores similar to those of controls [4].

The low levels of antidepressant use in VS patients are interesting considering what is known for low-grade glioma [33] and meningioma [43], where the rate of antidepressant use is distinctly increased after surgery. For low-grade glioma, the increased use of antidepressants is presumably related to the higher morbidity and mortality. For meningiomas, the reason is more elusive but could be related to that the outcome after VS surgery is generally more predictable, as meningiomas have a higher degree of variance concerning tumor location and symptoms and can cause seizures or cognitive symptoms. Meningioma surgery is also associated with a higher risk for complications than VS surgery [1, 5].

Sedatives are mainly prescribed for symptoms of anxiety and sleep disorders. They provide short-term symptom relief but have the potential for addiction, both through psychological and pharmacological mechanisms [46]. In addition, long-term use can have detrimental effects on health [41]. The baseline level of 4.3% for controls 2 years before the index date was in line with previous reports of sedative use in the general population [2, 30]. The rate of sedative use for VS patients at the index date was 6.9% for patients and 3.8% for controls. Thus, there was a trend towards increased sedative use for VS patients at the index date in our material, but no significant difference was found neither at 2 years before surgery, at the index date, or at 2 years after surgery.

Our study of a comparable population undergoing meningioma surgery during a similar time period demonstrated a clear peak around the time of surgery and a slight increase in long-term sedative use [43]. The expected brief, but significant, increase in sedative use for patients a few weeks after the index date did not cause long-term increased use in patients with VS.

### Return to work

The baseline level of sick leave for VS patients did not differ from the level for controls at 1 year before surgery (12% vs 10%). After surgery, more than half of VS patients had returned to work after 6 months, 66% after 1 year, and 75% after 2 years, while sick leave for controls remained stable. This contrasts with a 2009 study comparing surgery to SRS for VS patients. Among 22 non-retired surgically treated patients, 64% (14/22) were working 2 years after surgery. The SRS group had a lower RTW rate of 61% (27/44) but was older and had more retirements. Besides a small sample size, the study included patients above the age of 60, and it is not entirely clear how sick leave was defined and measured, potentially explaining the disparity in our data [20].

A 2002 questionnaire study of unilateral non-neurofibromatosis VS comparing VS surgery to SRS reported that in the surgically treated group of 110 included patients, 67% had returned to work at the time of follow-up. Comparisons to our material are somewhat difficult as the time from treatment to follow-up for surgical patients was not clearly stated and RTW was self-reported. Also, patients older than 60 were not excluded (the mean age was 61 years), presumably contributing to a relatively low RTW rate [27].

As with drug use, the rate of sick leave after VS surgery in our material is strikingly low compared to meningioma patients during the same time in the same region [42]. Compared to meningioma patients, VS patients appear less affected by their disease before surgery and the risk for complications affecting the ability to work in this patient group is lower [1, 5].

A large VS may compress the cerebellum and brainstem rather than the cerebrum and therefore be less inclined to cause cognitive symptoms. It has been shown for stroke patients that cortical neurological symptoms (such as language), but not location per se, are negatively correlated with RTW [47]. This observation could be relevant also when considering the risk of sick leave among VS patients compared to supratentorial disorders.

We observed that the rate of sick leave depends heavily on the timing of assessment and can be radically different at 1, 3, or 6 months after surgery. Therefore, comparisons between studies are difficult if the studied time points are not clearly defined. Many studies of sick leave and disability concerning VS surgery do not take this into consideration. This is a limitation in cross-sectional studies where patients are assessed at various time points. We recommend that prior to planning future studies, care should be taken to define the time point in relation to surgery for each measurement to be made, and researchers may find the figures presented in this manuscript useful in their planning.

In the multivariable regression model, we found that having more sick leaves in the year preceding surgery predicted not having returned to work 2 years after surgery. This is reasonable, considering that a history of sick leave has been shown to decrease the probability of RTW [15, 18] and that previous sick leave predicts future episodes of sick leave [12, 31].

An interesting finding was that sedative use at the index date was negatively associated with RTW at 2 years after surgery. Sick leave for psychological causes (such as depression or anxiety) is not uncommon and is associated with an increased risk of persisting into long-term sick leave [23]. Benzodiazepine use has been shown to correlate with unemployment [28]. The use of benzodiazepine also increases the likelihood for episodes of sick leave, although it is not clear by what mechanism [11]. Although sick leave has been associated with depression [28], the use of antidepressants did not predict sick leave in our study. Based on our findings, one may speculate if minimizing the use of sedatives around the time of surgery can increase the rate of RTW. However, further studies are needed to clarify the cause–effect relationship of this finding. Finally, we found that new-onset neurologic deficit after surgery reduced the probability of RTW at 2 years postoperatively. Because this variable is potentially modifiable, this finding is a reminder of the importance to minimize the risks of VS surgery.

### Strengths and limitations

The present study is an observational study. Inherently, this limits our ability to draw causal conclusions about the relationship between surgical treatment of VS and the studied outcome measures. We are also limited by the variables collected by the registries, where interesting variables such as surgical approach were not included. VS treated with upfront radiotherapy without histopathological verification would be interesting but were also not included in the data of the present study. Other variables, although present, were crude. For instance, the variable “new neurological deficit” may include both predictable loss of hearing, facial nerve injury, and/or unexpected neurological deterioration. Another limitation is that we had no access to which other interventions, for instance, psychotherapy, were used to treat depression.

A strength of the study is the nationwide registry, significantly reducing many forms of selection bias. The 333 patients included constitute a relatively large population considering that VS is rare, with a yearly incidence of around 1–2 per 100,000 inhabitants per year and that only a subset of VS patients requires surgery [40, 44]. Another strength of our study is that the avoidance of response bias, as the reporting to the national registries, has been mandatory. This is important because outlier groups can have aberrant response patterns, and nonresponse bias can severely skew results if the response rate is below 80%.

We have not compared VS surgery to other treatment forms, such as observation or SRS. Since we studied only surgically treated patients, it is likely that large and symptomatic VS are more common in our material than in a wider cohort of VS patients. VS with a radiological, but no histological diagnosis, however, constitutes a continuum of entities from miniscule uncertain findings to large tumors, making these hard to define and study.

From an international perspective, Sweden has a generous policy regarding the degree and length of sick leave. This may affect the external validity of the results in this article. To compensate for this, we have included matched controls in the study.

## Conclusions

The use of antidepressants and sedatives, and the rate of sick leave, was similar between VS patients and controls at baseline preoperatively. No differences were found between patients and controls regarding antidepressant and sedative use at the time of surgery or at 2 years after surgery. However, at 2 years after surgery, considerably more patients than controls were on sick leave. The risk for long-term sick leave increased in patients with more sick leave during the year preceding surgery, in patients using sedatives, and if surgery resulted in new neurological deficit(s).

## Supplementary Information

Below is the link to the electronic supplementary material.

Supplementary file1 (DOCX 42 KB)

## Abbreviations

ATC: Anatomic therapeutic chemical
MDD: Major depressive disorder
RT: Radiotherapy
RTW: Return to work
SBTR: Swedish brain tumor registry
SRS: Stereotactic radiosurgery
VS: Vestibular schwannoma
WHO: World Health Organization
