# Supplementary material for: Depression and ability to work after vestibular schwannoma surgery: a nationwide registry-based matched cohort study on antidepressants, sedatives, and sick leave
Source: Acta Neurochir (Wien). 2021 May 7;163(8):2225–35. doi: 10.1007/s00701-021-04862-8 (PMC8270857; doi:10.1007/s00701-021-04862-8)
Supplement: Supplementary file 1 — Supplementary file1 (DOCX 42 KB) [file 701_2021_4862_MOESM1_ESM.docx]

**Supplementary figure 1. Flow-chart of patient selection**

**Patients**

**Controls**

**Exclusion**

Surgically treated VS patients identified in SBTR

n = 346

Missing data or region with <80% registration rate

n = 13

**Control population to the full cohort:
n = 1662**

**Full cohort:**

**n =333**

Age > 60 years
n= 90

No sick-leave on the day of surgery

n = 36

**Control population to the RTW cohort:**

**n=1025**

**RTW cohort**

**n = 206**
